# Supplementary material for: Targeting the Notch-regulated non-coding RNA TUG1 for glioma treatment
Source: Nat Commun. 2016 Dec 6;7:13616. doi: 10.1038/ncomms13616 (PMC5150648; doi:10.1038/ncomms13616)
Supplement: Supplementary Information — Supplementary Figures 1-11 [file ncomms13616-s1.pdf]

a **b**

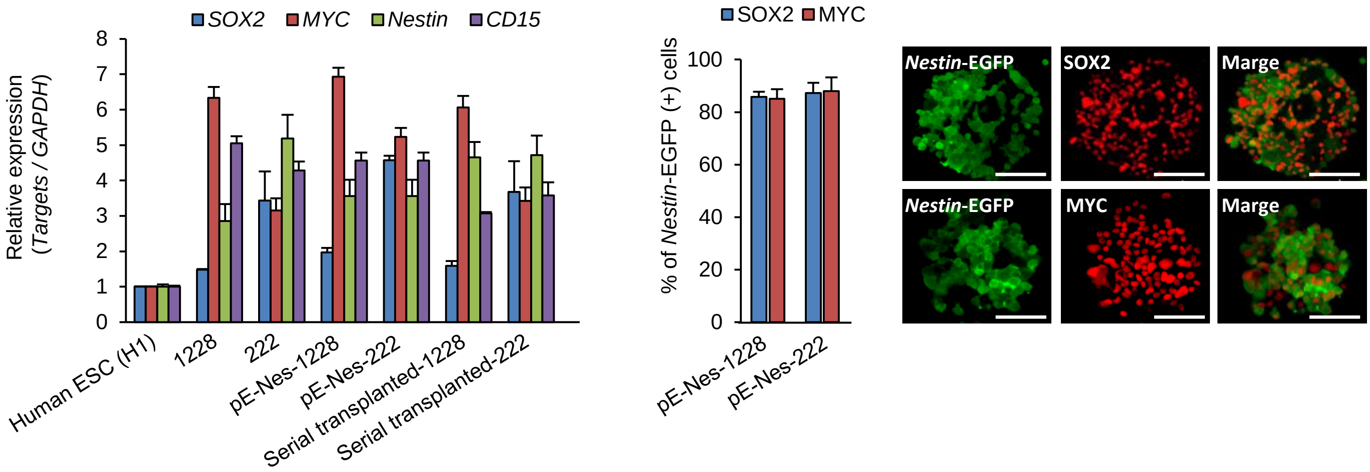

c

Line	Expression levels of stemness markers		Expression levels of differentiation markers		Tumorigenicity by limiting dilution assay		
					Number of cells injected		
	SOX2	CD15	GFAP	TUJ1	$1x10^2$	$1x10^3$	$1x10^4$
1228	81±4.5	88±7.1	5.4±0.7	4.2±1.3	5/5 (100)	3/3 (100)	5/5 (100)
222	91±6.3	89±6.2	3.3±0.2	1.3±0.7	3/3 (100)	3/3 (100)	3/3 (100)
pE-Nes-1228	79±4.5	75±3.9	5.5±0.9	6.5±1.4	3/3 (100)	3/3 (100)	3/3 (100)
pE-Nes-222	90±6.4	88±7.8	4.2±1.2	2.4±0.8	3/3 (100)	3/3 (100)	3/3 (100)

Supplementary Figure 1. Characterization of stemness and tumorigenic features of GSCs
(**a**) Expression levels of stemness markers (*SOX2*, *MYC*, *Nestin* and *CD15*) in GSCs (1228, 222, pE-Nes-1228 and pE-Nes-222) and serially transplanted-GSCs (1228 and 222). Relative expression level compared to that in human ES cell (H1) is indicated in Y-axis. Error bars indicate s.d. (**b**) Frequencies of EGFP-positive (*Nestin*-positive) cells in SOX2- and MYC-positive cells in pE-Nes-222 and pE-Nes-1228. SOX2 and MYC positive cells were counted in multiple spheres and calculated (left graph). Image of pE-Nes-222-GSC is shown (right panel). Error bars indicate s.d. Bar, 100 µm. (**c**) Summary of GSC characteristics. Expression level of stemness and differentiation markers was assessed by immunostaining. Expression levels of each marker were evaluated by frequencies (%) of immunopositive cells among GSC neurospheres. A total of 100 cells per line were counted in triplicate. Tumorigenicity of the GSCs was evaluated by limiting dilution assay (transplanted with $1x10^2$, $1x10^3$ and$1x10^4$ cells) *in vivo*. The tumorigenic rate (%) is shown in brackets.

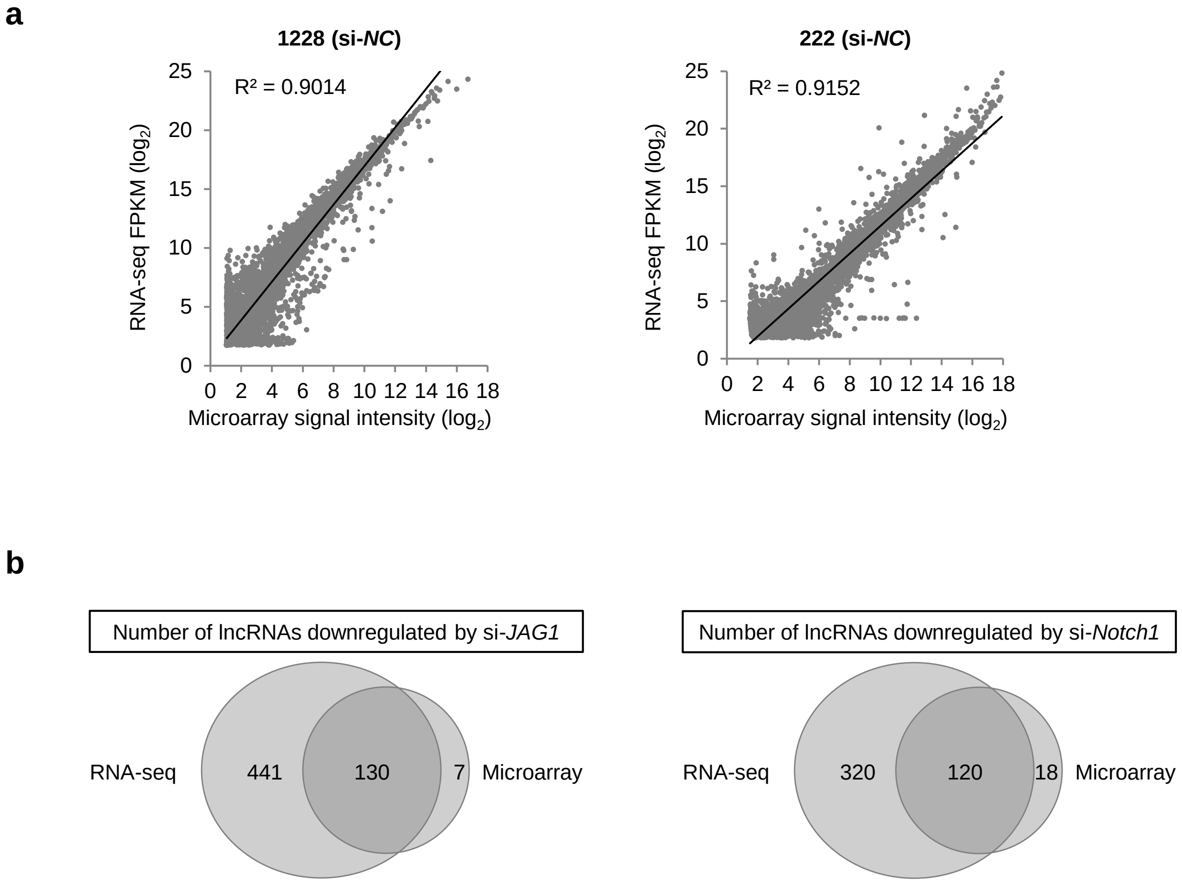

Supplementary Figure 2. Comparison between RNA-seq and microarray analyses in si-*JAG1* and si-*Notch1* treated GSCs
(**a**) Scatterplot comparison of global expression profiles of GSCs (1228 and 222) by using RNA-seq and microarray technology. Relationship between RNA-seq data and microarray data is estimated by Regression analysis. (**b**) Venn diagram depicts the numbers of downregulated lncRNAs identified by RNA-seq and microarray technology. List of lncRNAs were summarized in Supplementary Date 1 and 2.

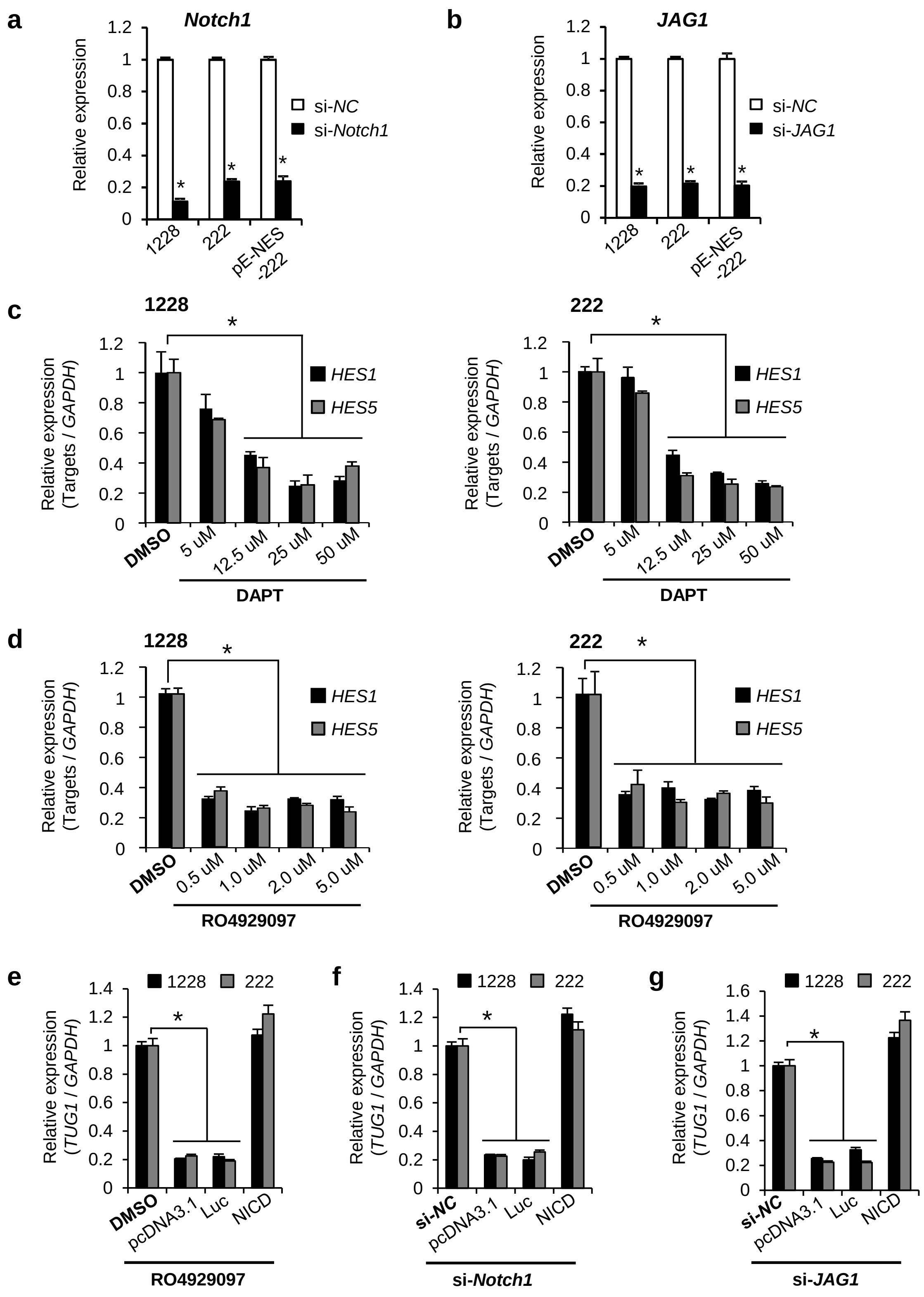

Supplementary Figure 3. Effects of Notch signaling on *TUG1* expression in GSCs

(**a**, **b**) Expression changes of indicated genes in GSCs treated with siRNA against *Notch1* (**a**) or *JAG1* (**b**). Values are indicated relative to abundance in si-*NC*-treated cells.*, *P*<0.01, Student's *t*-test. (**c**, **d**) Expression levels of *HES1* and *HES5* in GSCs treated with different concentrations of DAPT (**c**) or RO4929097 (**d**). Values are indicated relative to abundance in DMSO-treated cells. *, *P*<0.01, Kruskal-Wallis analysis. (**e-g**) Effect of NICD overexpression on *TUG1* expression. Expression levels of *TUG1* in GSCs treated with either RO4929097 (e), siRNA against *Notch1* (**f**) or *JAG1* (**g**). Empty vector (pcDNA3.1) and plasmid vectors expressing luciferase (Luc) were used for negative controls. Values are indicated relative to abundance in DMSO or si-*NC*-treated cells. *, *P*<0.01, Kruskal-Wallis analysis. For all experiments data, error bars indicate s.d. (n=3).

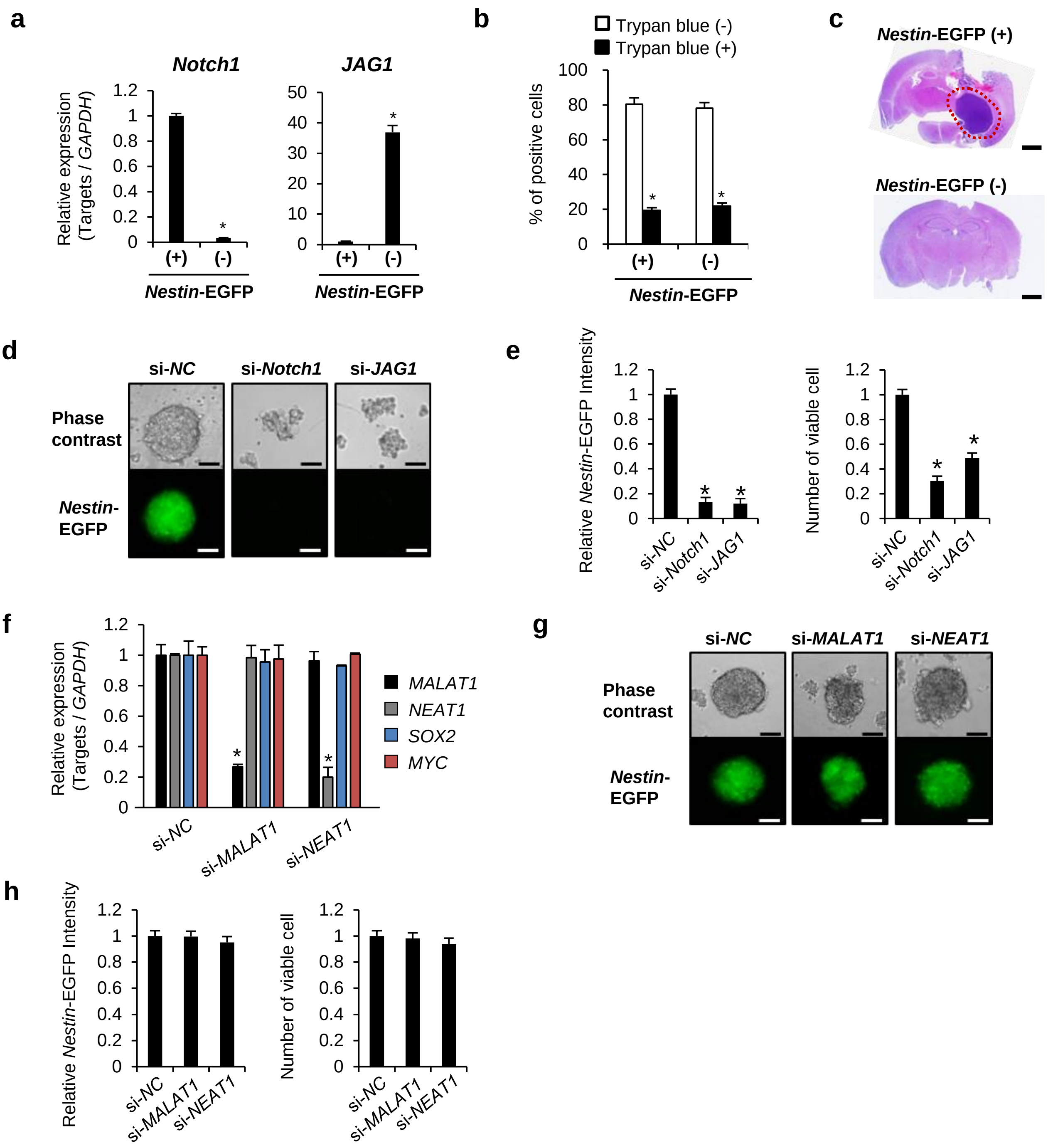

Supplementary Figure 4. Phenotypic characterization of GSC-pE-Nes

(**a**-**c**) Characterization of EGFP-positive (i.e. *Nestin*-positive) and EGFP–negative (i.e. *Nestin*-negative) GSCs. (**a**) Expression levels of *Notch1* and *JAG1* in *Nestin*-EGFP-positive and -negative cells. Relative expression level compared to that in *Nestin*-EGFP (+) is indicated on the Y-axis. *, P<0.01, Student's t-test. (**b**) Number of viable cells in EGFP-positive and -negative GSCs was assessed by trypan blue staining. *, P<0.01, Student's t-test. (**c**) *Nestin*-EGFP-positive and -negative cells were transplanted intracranially to NOD/SCID mice. Representative image (4 weeks post-transplantation) of HE staining is shown. Tumor areas are surrounded by red dotted line. Bars, 1 mm. (**d**) Effects of siRNA-mediated inhibition against *Notch-1* and *JAG1*. Phase-contrast and *Nestin*-EGFP images of GSC-pE-Nes-222 transfected with the indicated siRNAs are shown. Bars, 100 µm. (**e**) Relative intensity of *Nestin*-EGFP (left) and number of viable cell (right) to si-*NC* control were analyzed. Viable cells were assessed by trypan blue staining. *, P<0.01, Student's t-test. (**f**) Expression levels of stemness markers (*SOX2* and *MYC*) in GSCs after either si-*NC*, si-*MALAT1* or si-*NEAT1* treatment. Y-axis indicates relative expression level compared to that in si-*NC*-treated cells. *, P<0.01, Kruskal-Wallis analysis. (**g**) Phase-contrast and *Nestin*-EGFP images of pE-Nes-222 after treatment with either si-*NC*, si-*MALAT1*, or si-*NEAT1*. Bar, 100 µm. (**h**) Intensity of *Nestin*-EGFP (left) and number of viable cells (right) to si-*NC* control were quantified. Viable cells were assessed by trypan blue staining. For all the experimental data, error bars indicate s.d. (n=3).

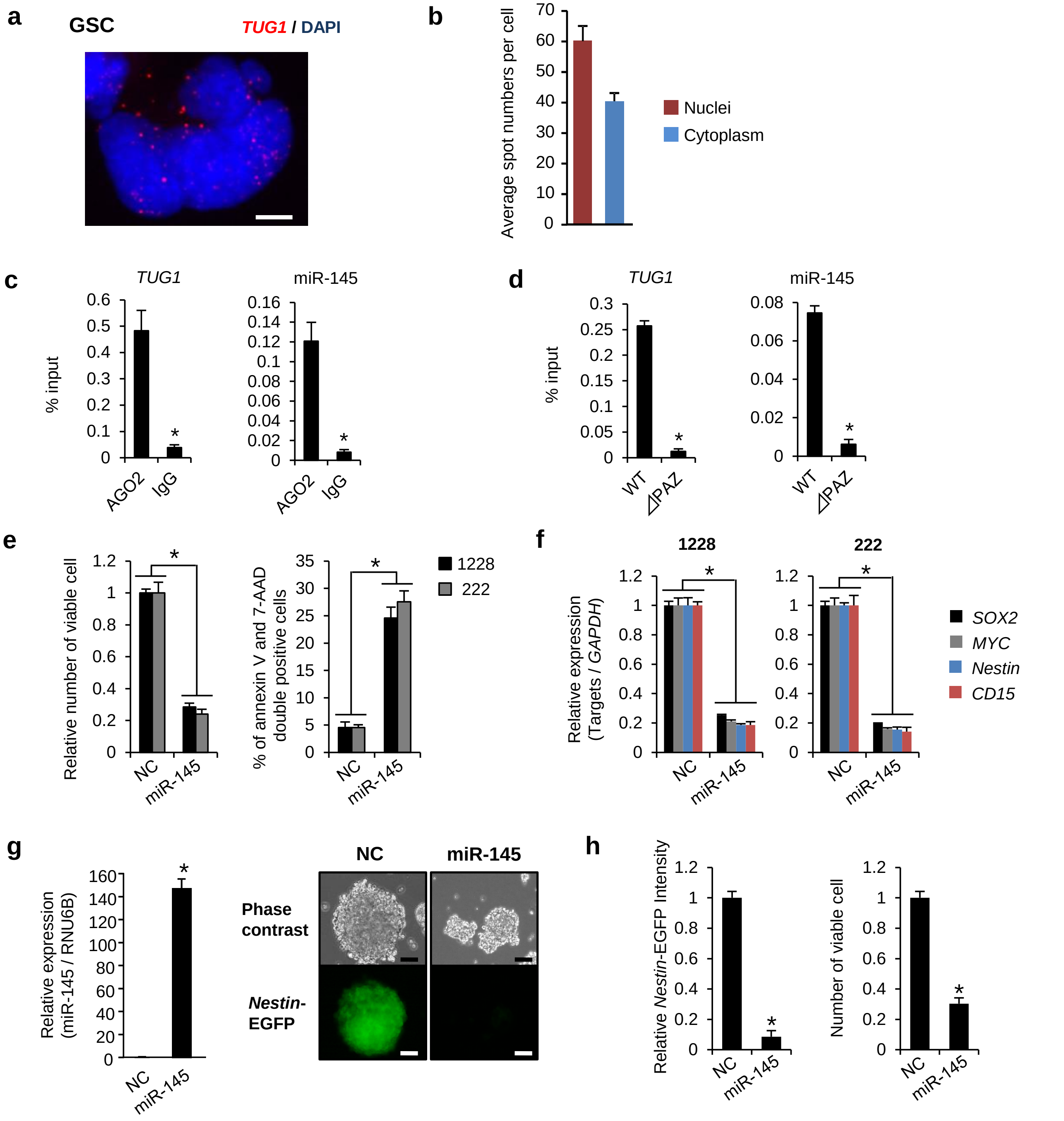

Supplementary Figure 5. Interaction between *TUG1* and miR-145 and its biological significance
(**a**) RNA-FISH analysis of *TUG1* (red) in GSCs. Nuclei are stained with DAPI. Bar,10 μm. (**b**) Ratio of subcellular localization of *TUG1* in GSCs. Multiple GSC lines are examined. Error bars indicate s.d. (**c**) RIP analysis of *TUG1* and miR-145 using an antibody against AGO2 or IgG. Enrichment of *TUG1* and miR-145 are expressed as a percentage of input RNA. (**d**) GSCs were transfected with expression vectors encoding either Flag-AGO2 (WT) or Flag-AGO2 lacking PAZ domain (ΔPAZ). RIP analysis of *TUG1* and miR-145 was performed by using an antibody against Flag. Enrichment of *TUG1* and miR-145 are expressed as a percentage of input RNA. (**e**) The number of viable cells (left) and apoptotic cells (right) among GSCs treated with a precursor molecule of miR-145 (miR-145) or negative control miRNA precursor (NC). Viable cells were assessed by trypan blue staining. Apoptotic cells were counted by FACS analysis with 7-AAD and PE Annexin V staining. (**f**) Effect of miR-145 on expression of the stemness-associated genes (*SOX2*, *MYC*, *Nestin* and *CD15*). Y-axis indicates relative expression level compared to that seen in NC-treated cells. (**g**, **f**) Effect of exogenous miR-145 on *Nestin* activity. (**g**) GSC-pE-Nes-222 were treated with a precursor molecule of miR-145 (miR-145) or negative control miRNA precursor (NC). Expression level of miR-145 (values are indicated relative to abundance in NC-treated cells, left). Phase-contrast and *Nestin*-EGFP images. Bar, 100 μm (right). (**h**) Intensity of *Nestin*-EGFP (left) and number of viable cells (right) compared to NC control were quantified. Viable cells were assessed by trypan blue staining. For all the experimental data, error bars indicate s.d. (n=3) *, *P*<0.01, Student's *t*-test.

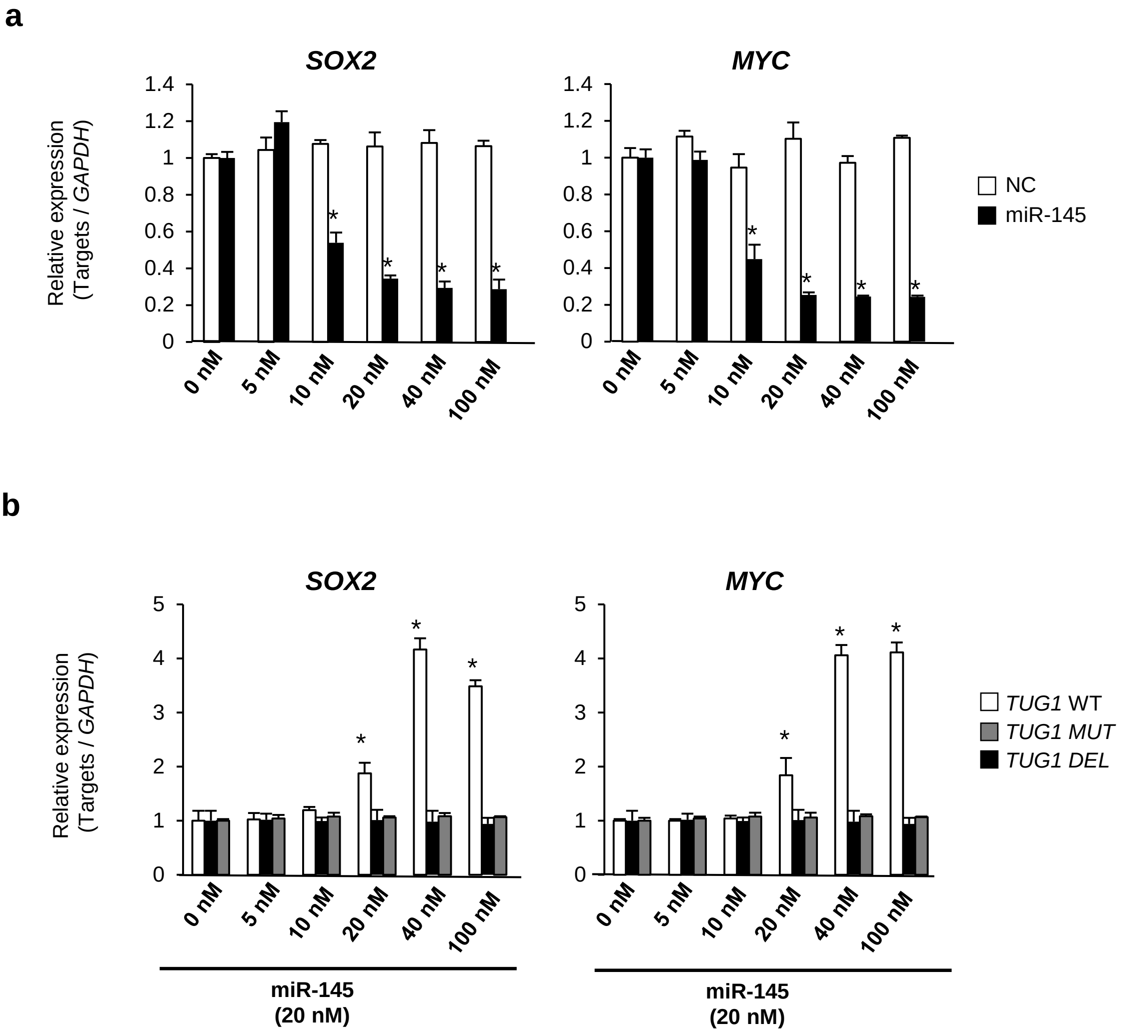

Supplementary Figure 6. Quantitative interaction between *TUG1* and miR-145
(**a**) Expression levels of *SOX2* and *MYC* in GSCs treated with the indicated doses of miR-145 or negative control miRNA precursor (NC). Values are indicated relative to abundance in non-treated cells (0 nM).*, *P*<0.01, Student's *t*-test. (**b**) Different concentrations of partial *TUG1* transcripts (1 to 2132 nucleotides), which contain the seed sequence of miR-145 (*TUG1* WT), mutated *TUG1* (MUT) or seed sequence-deleted *TUG1 (DEL) were added to* GSCs treated with miR-145 (20nM). Equivalent dose (20nM) of partial *TUG1* transcripts quenched miR-145 activities. Expression levels of endogenous *SOX2* and *MYC* were measured by qPCR. Values are indicated relative to abundance in non-treated cells (0 nM). *, *P*<0.01, Kruskal-Wallis analysis. For all experiments data, error bars indicate s.d. (n=3).

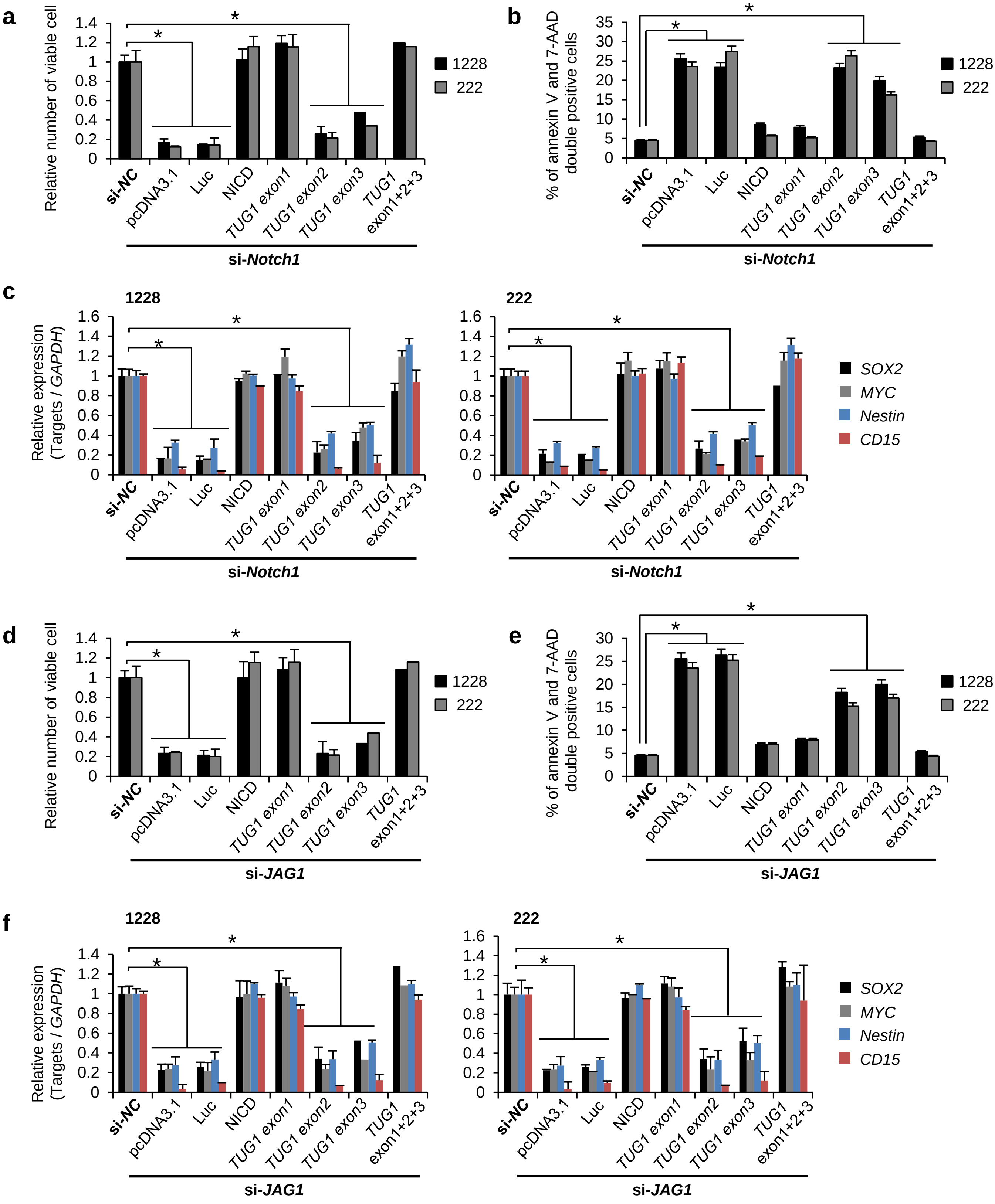

Supplementary Figure 7. Roles of each *TUG1 exon transcript* for the stemness features of GSCs
(**a**-**f**) Effects of *TUG1* overexpression on cell viability (**a, d**), apoptosis (**b, e**) and expression of the stemness-associated genes (*SOX2*, *MYC*, *Nestin* and *CD15*) (**c**, **f**) in GSCs treated with siRNA against *Notch1* (**a**-**c**) or *JAG1* (**d**-**f**). Plasmid vectors expressing indicated *TUG1* exons or NICD were added to GSCs. Empty vector (pcDNA3.1) and plasmid vectors expressing luciferase (Luc) were used for negative controls, while NICD overexpression (NICD) was used for a positive control for these experiments. Viable cells were assessed by trypan blue staining (**a**, **d**). The number of apoptotic cells were counted by FACS analysis with 7-AAD and PE Annexin V staining (**b**, **e**). Expression levels of stemness-associated genes were analyzed by qRT-PCR. Y-axis indicates relative expression level compared to that seen in si-*NC* -treated cells (**c**, **f**). Values are indicated relative to abundance in si-*NC*-treated cells. For all the experimental data, error bars indicate s.d. (n=3) *, P<0.01, Kruskal-Wallis analysis.

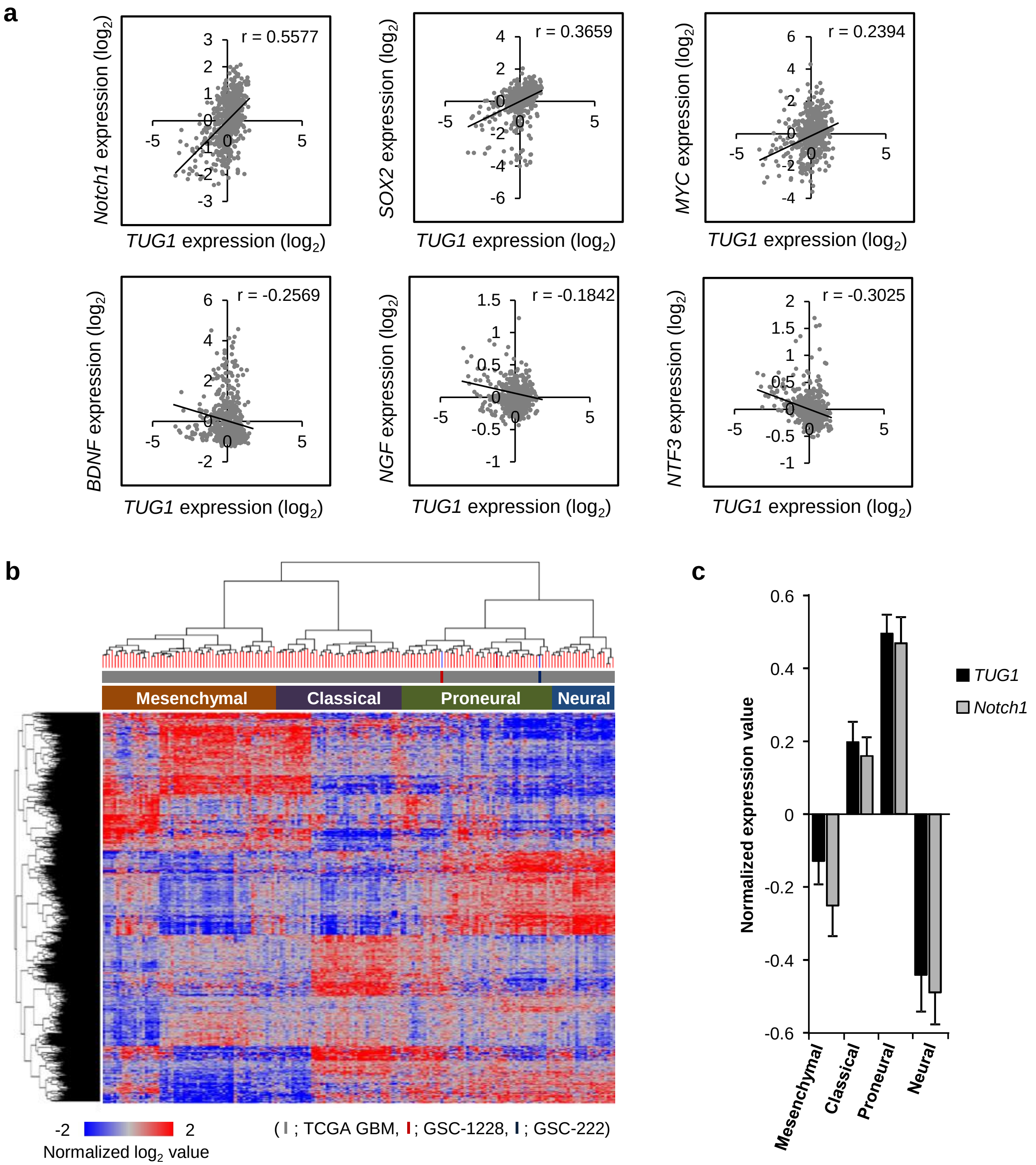

Supplementary Figure 8. Expression analysis of *TUG1* in clinical GBM samples
(**a**) Correlation between *TUG1*, *Notch1*, miR-145 target genes (*SOX2* and *MYC*), and *TUG1* target gene (*BDNF*, *NGF* and *NTF3*) expression in clinical GBM samples. Data were obtained from TCGA data sets. Each comparison is performed between the genes indicated on the X- and Y-axes, respectively. (**b**) Hierarchical clustering analysis of gene expression profiles using GBM data sets from the TCGA and the GSC lines (1228- and 222) studied here. Color corresponds to expression level as indicated in the log2-transformed scale bar below the matrix. Red and blue reflect high and low levels, respectively. On the X-axis, a grey bar indicates TCGA GBM samples. Red and blue bars indicate 1228- and 222-GSC, respectively. (**c**) Expression levels of *TUG1* and *Notch1* in TCGA GBM samples. Error bars indicate s.e.m.

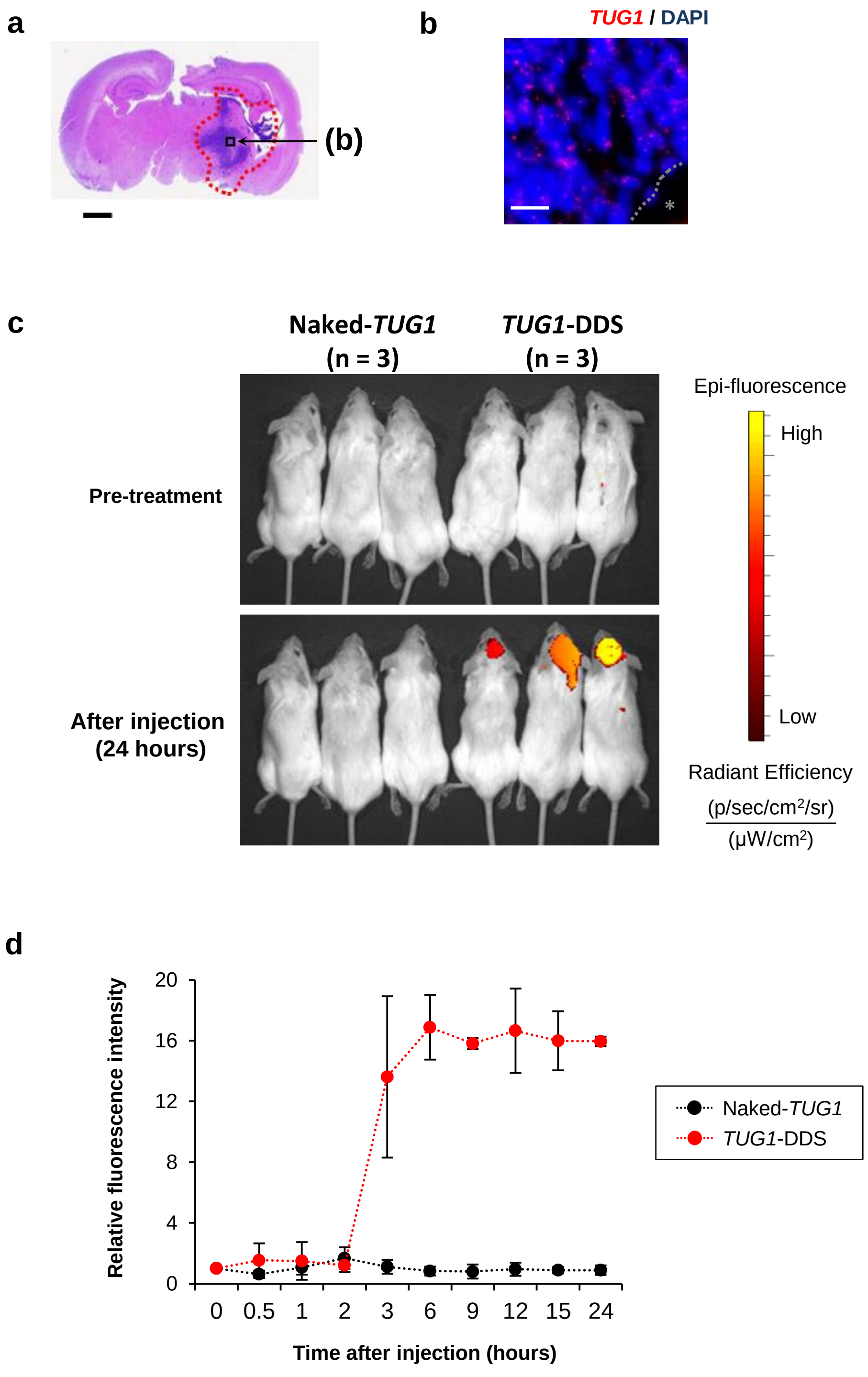

Supplementary Figure 9. Analysis of *TUG1* expression and *TUG1*-DDS accumulation in the mouse
(**a**) Representative image of HE staining of mouse xenograft after 30 days of transplantation. Tumor areas are surrounded by red dotted line. Bar, 1 mm. (**b**) RNA-FISH analysis of *TUG1* (red) in tumor cells around area **b** (perivascular region) in panel **a**. Nuclei were stained with DAPI. Asterisk indicates blood vessel. Bar, 20 mm. (**c**) Mice bearing brain tumors were intravenously administered with naked (without DDS) Alexa647 labeled ASO-*TUG1* (Naked-*TUG1*, n=3; left) or Alexa647 labeled *TUG1*-DDS (right, n=3). Fluorescence intensity was analyzed using an IVIS imaging system. (**d**) *TUG1*-DDS was stably accumulated in the tumors at least for 24 hours. Values are indicated relative to intensities at the point of pre-treatment (0 hour). Error bars indicate s.e.m.

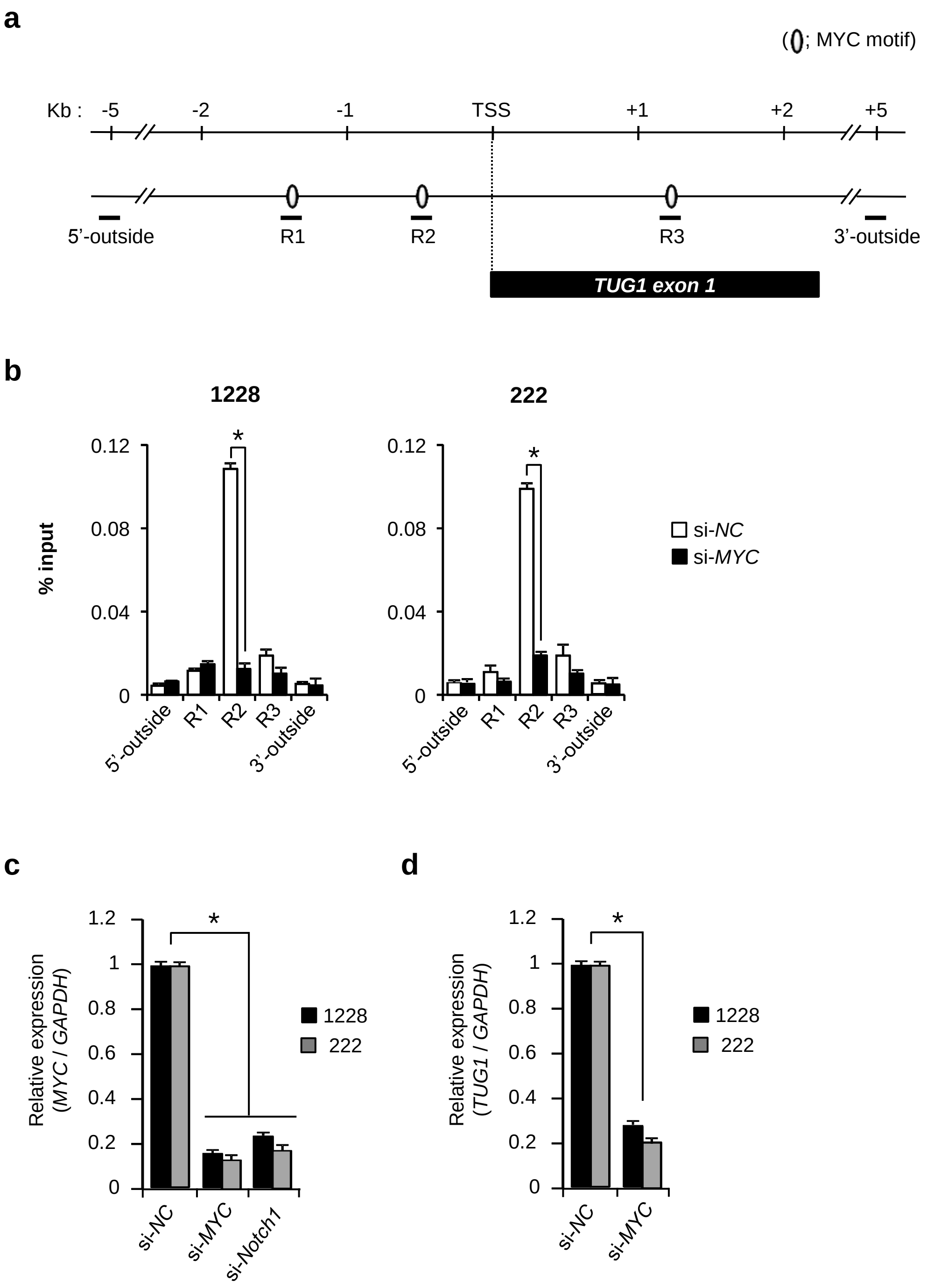

Supplementary Figure 10. MYC promotes *TUG1* expression in GSCs

(**a**, **b**) ChIP analysis of MYC in the upstream region of *TUG1* TSS. (**a**) Schematic diagram showing MYC motifs around the TSS of *TUG1*. Open circles indicate MYC motifs. (**b**) Enrichment of MYC in GSCs treated with either si-*NC* or si-*MYC*. Regions examined by ChIP analysis are indicated as 5'-outside, R1, R2, R3 and 3'-outside in **a**. Enrichment of MYC is expressed as a percentage of input DNA. *, *P*<0.01, Student's *t*-test. (**c**) Expression level of *MYC* in GSCs treated with siRNA against the indicated genes on the X-axis. Relative expression level to the siRNA-negative control (si-*NC*) is indicated on the Y-axis. *P*<0.01, Kruskal-Wallis analysis. (**d**) Expression changes of *TUG1* in GSCs treated with siRNA against *MYC*. Values are indicated relative to abundance observed in si-*NC*-treated cells. *, *P*<0.01, Student's *t*-test. For all experiments data, error bars indicate s.d. (n=3).

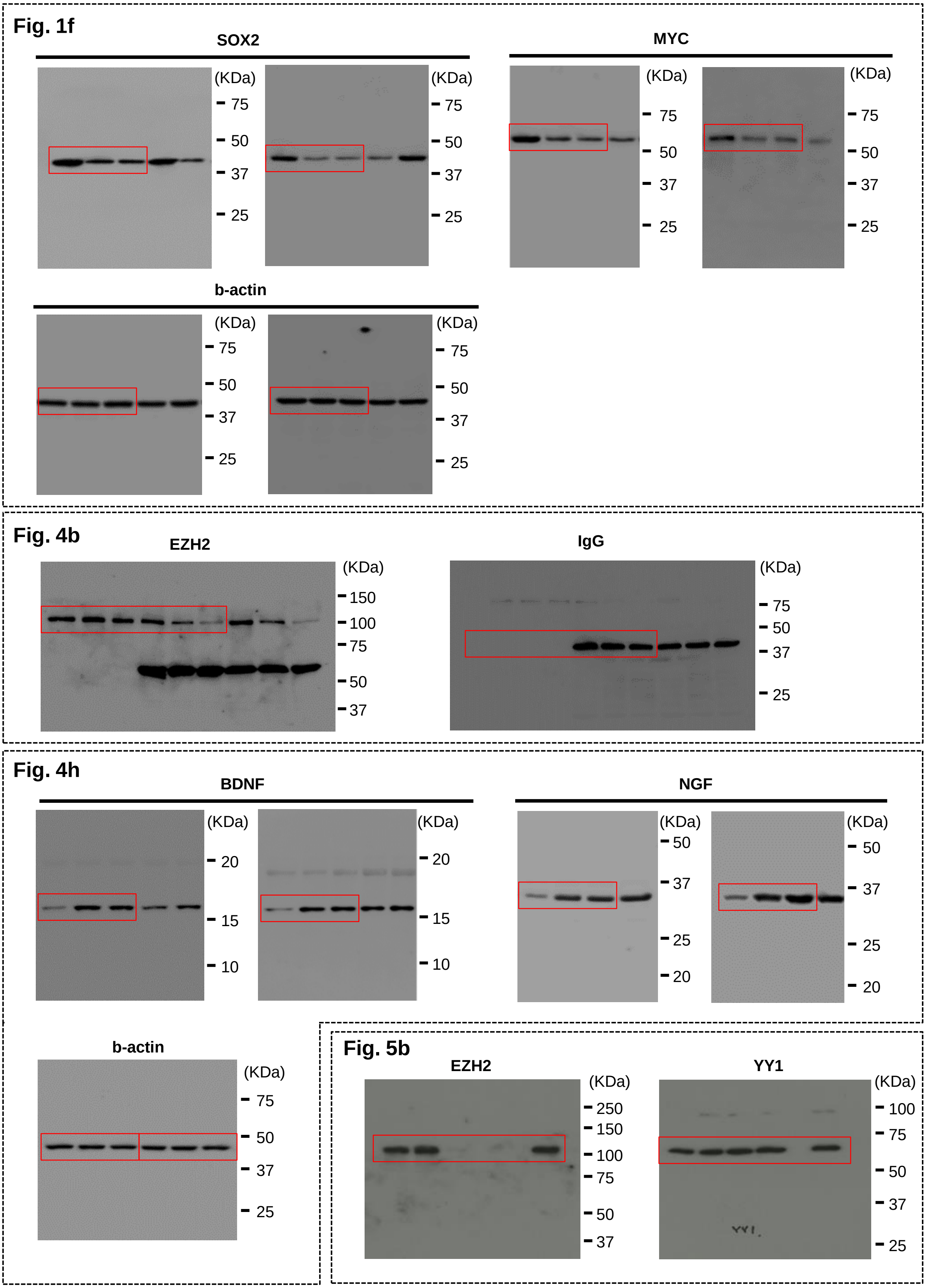

Supplementary Figure 11. The original immunoblot images utilized in this study
The red sections indicate blot results shown in the indicated figures.
